# Sensing Range Extension for Short-Baseline Stereo Camera Using Monocular Depth Estimation

**DOI:** 10.3390/s22124605

**Published:** 2022-06-18

**Authors:** Beom-Su Seo, Byungjae Park, Hoon Choi

**Affiliations:** 1 Intelligent Robotics Research Division, AI Research Laboratory, Electronics and Telecommunication Research Institute (ETRI), Daejeon 34129, Korea; bsseo@etri.re.kr; 2 School of Mechanical Engineering, Korea University of Technology and Education, Cheonan 31253, Korea; 3 Department of Artificial Intelligence, Chungnam National University, Daejeon 34134, Korea

**Keywords:** stereo camera, depth estimation, convolution neural network

## Abstract

This paper proposes a method to extend a sensing range of a short-baseline stereo camera (SBSC). The proposed method combines a stereo depth and a monocular depth estimated by a convolutional neural network-based monocular depth estimation (MDE). To combine a stereo depth and a monocular depth, the proposed method estimates a scale factor of a monocular depth using stereo depth–mono depth pairs and then combines the two depths. Another advantage of the proposed method is that the trained MDE model may be utilized for different environments without retraining. The performance of the proposed method is verified qualitatively and quantitatively using the directly collected and open datasets.

## 1. Introduction

Range sensors are required to recognize the surrounding environment for autonomous platforms, such as autonomous vehicles, service robots, and drones. LiDAR and stereo cameras are typical range sensors that provide distance measurements, among sensors that can be mounted on platforms. LiDAR is applied on various platforms because it has a long detection range without being affected by lighting changes. However, there are two constraints to using LiDAR on a platform. The first constraint is the size of the LiDAR sensor. When the size of the platform is small, it may be difficult to mount a LiDAR sensor because of its size. The second constraint is the LiDAR sensor needs a lot of power from the platform because it must generate signals actively. In the case of large platforms, such as autonomous vehicles, there is plenty of room to install a LiDAR, and there is no problem in supplying power to the LiDAR. However, small platforms, such as service robots and drones for small delivery, may not have enough room to install LiDAR. Furthermore, the size of the battery loaded on a small platform is small, and if a LiDAR is mounted, it will cause problems such as a shorter operation time and a narrower operation range.

Unlike LiDAR, it is easy to mount a stereo camera on a small platform because it can be made passively in a small size and measure distance. Moreover, RGB images provided by the stereo camera can be used for other purposes as well, such as object detection or tracking. However, stereo cameras have two drawbacks, different from LiDAR. First, although various methods have been proposed to overcome this by adaptively controlling exposure [1], they are affected by lighting changes. Second, the distance that can be measured by stereo cameras is shorter than that of LiDAR. The method for measuring the distance from a stereo camera is to calculate the disparity using stereo matching and use the characteristics of the stereo camera, such as focal length and baseline. The baseline is a value of the stereo camera that has the greatest influence on distance measurement. The longer the baseline is, the longer the distance that the stereo camera can measure. However, if the baseline is long, the advantage of the stereo camera over LiDAR—it is small and can be mounted on a small platform—disappears. Furthermore, even if the baseline is long, it will not detect the distance as accurately as LiDAR.

The disparity can be calculated more precisely as a way of improving the detection range without having a large baseline of the stereo camera. Recently, a convolutional neural network (CNN)-based stereo matching method such as PSMNet [2] was proposed. This method has two limitations although it can calculate the disparity precisely. First, a CNN-based stereo matching method requires many datasets to train the model and may not work well in environments different from the environment where the datasets used in the training were collected. Second, the platform must provide large memory capacity and computational power to use the CNN-based stereo matching method. Small platforms are limited in providing large memory capacity and computational power for precise disparity calculations.

In this paper, we use another approach to solve the short measurement range problem, which is a disadvantage of short-baseline stereo cameras (SBSC) used on small platforms for autonomous navigation. The proposed method combines the stereo depth (SD) of a limited range obtained with stereo matching from the SBSC and the CNN-based monocular depth estimation (MDE) [3] result. The CNN-based MDE can estimate the depth, even for a long-distance, with a monocular image only, but it has an issue that needs to be addressed when applying it to autonomous navigation. The MDE can only estimate the relative depth. A scale factor is additionally required to find the absolute depth value. A method of using the distance between the mounted position of the camera on the platform and the floor has been proposed to calculate the scale factor of MD, but there is the limitation that it cannot be used in unstructured environments [4].

To expand the SBSC’s sensing range, this paper proposes a method that uses the SD of a limited range obtained from SBSC and the MD obtained from MDE to calculate the scale factor of MD and then combines the SD and MD to make the proposed depth (fused Depth:FD).

It is possible to estimate the depth with SBSC over a long distance if the recommended method is employed. The mapping, localization, and motion planning capabilities of the small platform can be improved if the proposed method is applied to an autonomous navigation system. In the case of motion planning, for example, the proposed method facilitates the detection of obstacles over longer distances, enabling safer and more efficient movements. The performance of the proposed method was validated qualitatively and quantitatively using various open and directly collected datasets.

This paper is organized as follows. Section 2 introduces various CNN-based MDE methods and compares their performances. Section 3 explains the proposed method’s motivation and pipeline in detail. Section 4 introduces the experimental results obtained using the open and directly collected datasets, and Section 5 provides the conclusions and future works.

## 2. Convolutional Neural Network-Based Monocular Image Estimation

Structure-from-motion (SFM) is generally used to obtain the environment’s three-dimensional (3D) information using a monocular camera. SFM uses multiple images obtained from different positions as inputs. MDE, unlike SFM, can obtain the environment’s 3D information from only a single RGB image. In CNN-based MDE, a single image is used as an input, and a depth image that has the same size as the input image is output. Because CNN-based MDE can be applied to fields such as autonomous navigation, as well as augmented reality, various methods that can operate in real-time in edge devices have been actively proposed [4,5,6,7]. The aforementioned methods typically use a supervised approach with LiDAR-acquired ground truth. Some of them employ a self-supervised technique in the absence of ground truth [8]. Recently, some approaches use the transformer [9,10,11].

**Figure 1:**
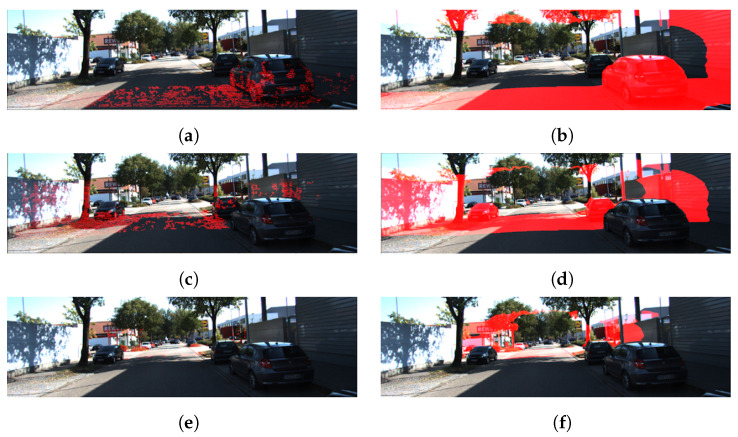
Depth estimation results of the KITTI test dataset for the AdaBins model trained using the KITTI training dataset (left: ground truth, right: depth estimation result). The regions shown in red in the images indicate the point groups corresponding to certain depth ranges. (**a**,**b**) 5–10 m, (**c**,**d**) 10–20 m, (**e**,**f**) 20–40 m.

However, there is an issue when the MD obtained from CNN-based MDE is used in mapping, localization, and motion planning for autonomous navigation. When the camera parameters and environment are similar to those of the dataset used to train the CNN model for MDE, the CNN model can estimate the depth accurately. In the meantime, if the environment and camera are not the same as in the training dataset, the absolute distance in the depth estimation result is inaccurate, though the relative distance is accurate. Using AdaBins [7], a method that shows the best performance among the recently proposed CNN-based MDE methods, we confirmed that this issue exists. When an AdaBins model trained using the KITTI training dataset [12] is applied to the KITTI test dataset, it is found that the AdaBins model can estimate the depth accurately, even for a long distance (40 m). Figure 1 shows the depth estimation results of the KITTI test dataset for the AdaBins model trained with the KITTI training dataset. In Figure 1, the red regions indicate the point groups corresponding to certain depth ranges (5–10 m, 10–20 m, and 20–40 m). As shown in the figure, the AdaBins model estimates depths up to about 40 m.

**Figure 2:**
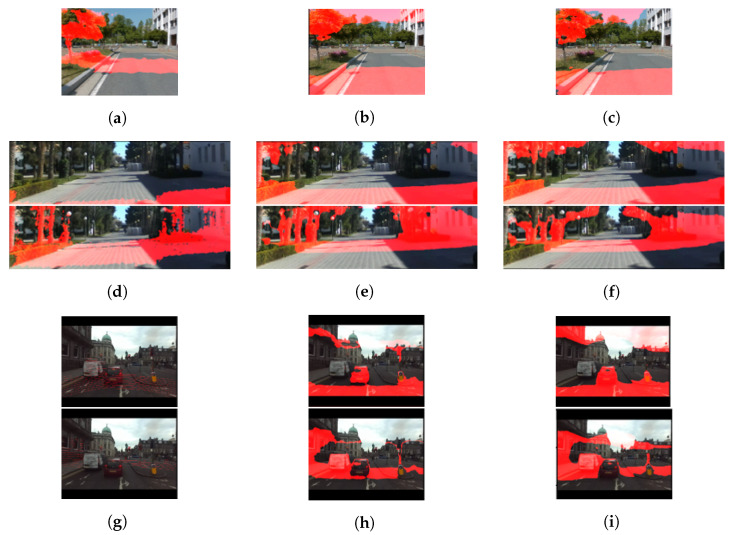
Results of using the models (BTS, AdaBins) trained with the KITTI training dataset for depth estimation of other datasets (from left to right: ground truth, BTS depth result, AdaBins estimation result). The red dots and regions in the ground truth indicates a point group existing in the depth estimation range of the stereo camera used to collect the dataset. The red regions in the BTS and AdaBins depth estimation results indicate the point groups corresponding to depth ranges. (**a**–**c**) CNU dataset (5 to 10 m), (**d**–**f**) UASOL dataset (upper: 5 to 10 m; lower: 10 to 20 m), (**g**–**i**) RADIATE dataset. (upper: 5 to 10 m; lower: 10 to 20 m).

However, the depth estimation results are inaccurate when the model trained with the KITTI training dataset is applied to a different environment or a different camera. Figure 2 shows the results of applying two well-known models (BTS [5], AdaBins [7]) trained with the KITTI training dataset to the depth estimation of datasets (CNU(Chungnam National University), UASOL [13], RADIATE [14]) collected using different cameras in different environments. The CNU dataset was collected using Intel RealSense D435 (baseline: 50 mm) on a university campus in South Korea. The UASOL dataset [13] was collected using ZED (baseline: 120 mm) on a university campus in Spain. For the RADIATE dataset [14], the same camera as the UASOL dataset was used, but the images were captured in urban and suburban areas in the United Kingdom under various lighting and weather conditions. As shown in Figure 2, the depth estimation results of the models trained using the KITTI training dataset are quite different from the actual results. In Figure 2, the red dots and regions indicate a point group existing with the depth estimation range of the stereo camera used to collect the dataset: a 10 m region for the CNU dataset and a 20 m region for the UASOL and RADIATE datasets, respectively.

To expand the sensing range of the SBSC using the off-the-shelf approach without a process of retraining the MDE model again, the scale factor of MD must be estimated first. In the next section, we describe the method for estimating the MD’s scale factor without additional training of the model and explain the method of combining the SD obtained using this and the MD of a limited range obtained from the SBSC.

## 3. Sensing Range Extension

### 3.1. Overview

The scale factor of MD must be estimated first to expand the recognition range of SBSC without additional training using the MDE model. Assuming that the scale factor can be represented in some form of function regardless of environment or camera, it is possible to map the relative depth value inferred from the MD to the absolute value. Furthermore, if an appropriate parameter of an appropriate scale factor function can be found using the proposed method, the MDE model can be applied to a different environment and camera without new training whenever the environment or camera changes.

**Figure 3:**
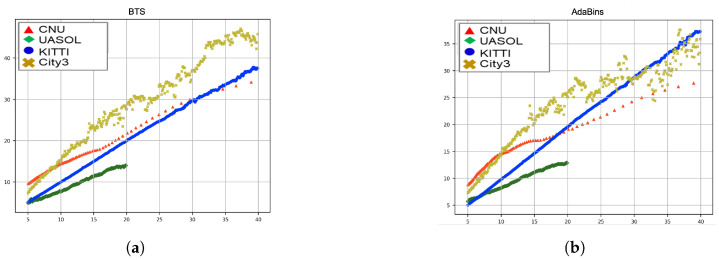
Mono depth estimation results for KITTI, CNU, RADIATE, and UASOL datasets using three models. Each model trained using KITTI datasets. (**a**) BTS, and (**b**) AdaBins.

To confirm that the scale factor can be represented in some form of function and only the parameter varies depending on the environment and camera, we analyzed the results using the MDE models trained with the KITTI training dataset to estimate the depth of other datasets. Figure 3 shows graphs comparing the depth of other datasets estimated by the model trained using the KITTI training dataset and the depth obtained using LiDAR or stereo matching. In the figure, the *x*-axis represents the actual distance that calculated the depth using LiDAR or stereo matching. The *y*-axis represents the mean of MD corresponding to the actual distance. In the case of KITTI and RADIATE datasets, the actual distance was calculated using LiDAR, and in the case of CNU and UNASO datasets, the actual distance was obtained using stereo matching. The actual distance measurement range using stereo matching is shorter than that of LiDAR because of the stereo camera’s characteristics. The estimated MD of the KITTI test dataset is found to be almost the same as the actual depth because the environment and camera learned are identical. However, the estimated MD of other datasets is very different from the actual depth. Moreover, the MD obtained through the MDE model and the actual depth have strong linearity. This linearity is common in BTS and AdaBins models. Therefore, the scale factor can be represented as a linear function, and it is possible to estimate the linear function’s parameter using the SD and MD estimated by the MDE mode.

**Figure 4:**
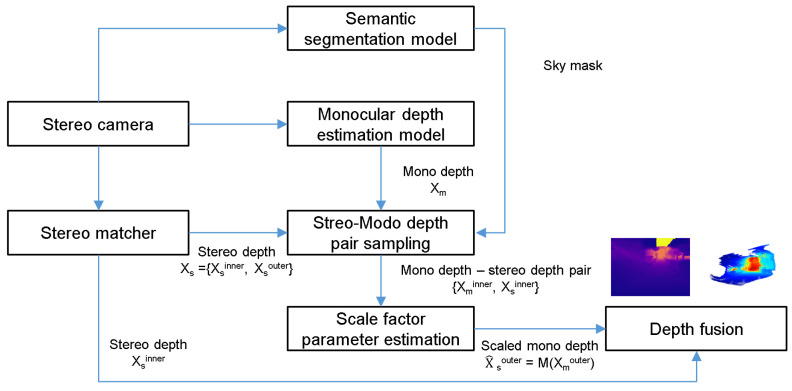
Pipeline of the sensing range extension method.

The process of expanding the recognition range of SBSC using the CNN-based MDE model proposed in this paper as shown in Figure 4 is as follows.

1. The SD depth ($X_{s}$) is estimated from the stereo camera.
2. MD ($X_{m}$) is estimated using the MDE model.
3. SD and MD pairs {$X_{m}^{inner}$, $X_{s}^{inner}$} are sampled in a reliable depth range.
4. The scale factor’s parameter (*M*) is estimated.
5. The scale of the MD outside of the reliable depth ($X_{m}^{outer}$) is estimated using the scale factor.
6. The SD depth and the scale factor-considered MD are combined to create the expanded depth (FD).

Along with the proposed method, we also used a method removing incorrect depth values existing in the sky region. Because of the lack of texture, ghosts appear in the sky region. These ghosts appear as unknown obstacles, which may cause problems with accurate environment recognition. To remove them, a CNN model for semantic segmentation is used. The depth value of a pixel that belongs to the sky region is not used to create MD–SD pairs.

### 3.2. Stereo-Mono Depth Pairing

As shown in Figure 3, the relationship between the actual depth and the MD estimated using the MDE model can be expressed as a linear function. Therefore, the SD of the limited range obtained through stereo matching and the MD estimated using the MDE model can be used to estimate the scale factor’s parameter. From the SD and MD, reliable SD–MD pairs must be created to estimate the scale factor’s parameter. In the KITTI training data, ground truths of the close area (5 m) are almost non-existent because of the configuration of the platform used when collecting the data. Therefore, MD estimation accuracy cannot be computed closer than 5 m. Furthermore, because of the structure of SBSC, there may be errors that are above a certain level in the depth values of a range greater than a certain distance (10 m) [14,15,16]. In the proposed method, a reliable range for creating pairs is first determined by considering the above and the camera’s performance and noise. The depth within the SD that has a value within the range ($d_{min}$ = 6.5 m to $d_{max}$ = 8.5 m) is used to create SD–MD pairs.

(1) $$\begin{array}{c} p\left(i,j\right)=\left(x_{m}\left(i,j\right),x_{s}\left(i,j\right)\right),\\ d_{min}\leq d\left(x_{s}\left(i,j\right)\right)\leq d_{max},\\ x_{m}\left(i,j\right)\in X_{m},x_{s}\left(i,j\right)\in X_{s},c\left(i,j\right)\neq c_{s}, \end{array}$$

In the above equation, $x_{m}\left(i,j\right)$ means the depth value corresponding to the pixel (*i*, *j*) in MD, and $x_{s}\left(i,j\right)$ means the depth value corresponding to the pixel (*i*, *j*) in SD. $d_{min}$ and $d_{max}$ are the minimum and maximum values of the reliable depths, respectively. c(i,j) refers to the class corresponding to the pixel in the semantic segmentation results, and $c_{s}$ is the class corresponding to the sky.

Algorithm 1 shows the abstracted process of the stereo-mono depth pairing. It uses the MD, SD, and semantic segmentation result (C) as an input to compute the stereo-mono depth pair. A mask operation is applied to improve the computation efficiency.

**Algorithm 1:** Stereo-Mono Depth Pairing**Input:** $X_{m}$, $X_{s}$, C, $d_{min}$, $d_{max}$ **Output:** P
 ${\mathrm{Idx}}_{mask}\leftarrow \left\{\left(i,j\right)|d_{min}\leq X_{s}\left[i,j\right]\leq d_{max}\right\}$
  $S_{mask}\leftarrow \left\{\left(i,j\right)|C\neq c_{s}\right\}$
 ${\mathrm{Idx}}_{mask}\leftarrow {\mathrm{Idx}}_{mask}\cap S_{mask}$
 $X_{s}\leftarrow X_{s}\left[{\mathrm{Idx}}_{mask}\right]$
 $X_{m}\leftarrow X_{m}\left[{\mathrm{Idx}}_{mask}\right]$
  $P\leftarrow (X_{m},X_{s})$
 **return** P


### 3.3. Scale Factor Parameter Estimation

**Figure 5:**
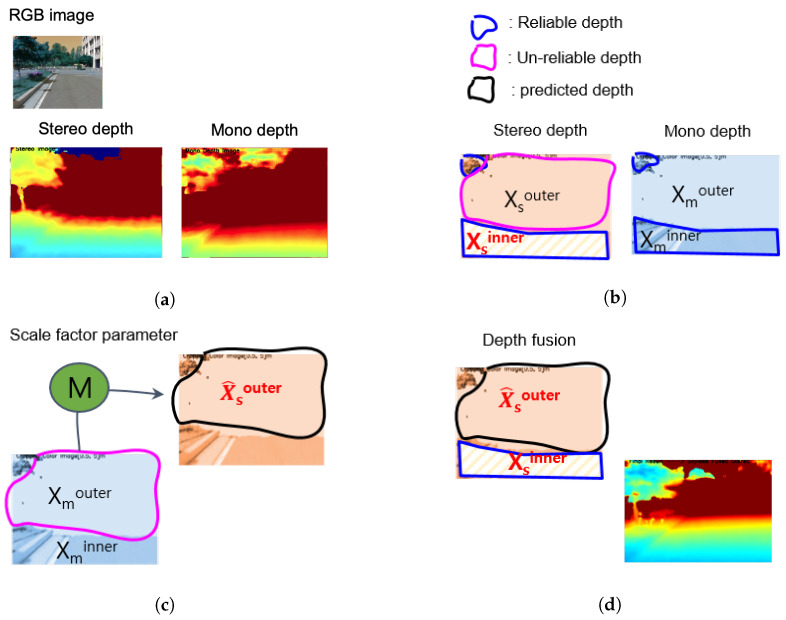
Detailed pipeline of the sensing range extension method. (**a**) RGB, Stereo and mono depths, (**b**) stereo-mono depth pair sampling, (**c**) outer stereo prediction using scale factor parameter model M, and (**d**) depth fusion.

There may be many noises in the SD–MD pairs because of the effect of the lighting or texture distribution in the RGB image. We used RANSAC, which is robust to outliers, to estimate the scale factor’s parameter. There is one issue when estimating the scale factor’s parameter using the RANSAC: although the number of data points of the SD–MD pairs within the reliable depth range is enormous, the distribution is not even. When the scale factor’s parameter is estimated using the unevenly distributed SD–MD pairs, a biased result is obtained. To prevent this, we sorted the SD–MD pairs based on the SD and performed bucketing (Algorithm 2). When bucketing, stereo depth values within a given distance range are gathered into a single stereo depth bucket $B_{s}$. The corresponding monocular depth values are also gathered into a single mono depth bucket $B_{m}$. RANSAC uses the mean depth of each bucket as an input to estimate the scale factor’s parameter. $N_{step}$ along with about 900 data points on average per bucket in the case of Figure 5.

**Algorithm 2:** Bucketing**Input:** P, $d_{min}$, $d_{max}$, $N_{step}$ $B_{s}=\left\{\right\}$, $B_{m}=\left\{\right\}$ $\delta =\left(d_{max}-d_{min}\right)/N_{step}$ **for***i* from 0 to $N_{step}-1$ **do**  $b_{s}\left[i\right]=\left\{x_{s}\left(i,j\right)|d_{min}+i\cdot \delta <=d\left(x_{s}\left(i,j\right)\right)<d_{min}+\left(i+1\right)\cdot \delta \right\}$  $b_{m}\left[i\right]=\left\{x_{m}\left(i,j\right)|x_{m}\left(i,j\right)\in p\left(i,j\right)\right\}$  $B_{m}\leftarrow B_{m}\cup b_{m}\left[i\right]$, $B_{s}\leftarrow B_{s}\cup b_{s}\left[i\right]$ 
**end for**
 
**return** 
$B_{s},B_{m}$


### 3.4. Scale Factor Parameter Update

Because the moving speed is not fast for most small platforms, the appearance of the surrounding environment does not change dramatically. It is possible to reduce the computational load used by the proposed method on small platforms if this condition is used when using the proposed method. The parameter estimated in the previous frame can be used when the appearance of the surrounding environment is similar to that of the previous frame, rather than estimating the scale factor’s parameter for every frame. For this, the following equation is used to check whether the parameter estimated in the previous frame can be used in the current frame.

(2) $$\begin{array}{c} e=\frac{\sum \left|M^{t-1}\left(x_{m}^{t}\left(i,j\right)\right)-x_{s}^{t}\left(i,j\right)\right|}{N}, \end{array}$$

The above equation obtains the error between the SD of the current frame and the scaled MD using the parameter estimated in the previous frame. $M^{t-1}$ is the parameter estimated in the previous frame, and *N* is the number of data points of the stereo MD–SD pairs in the current frame. If the value of *e* is not larger than the threshold value, the scale factor’s parameter is reused without estimating in the current frame. If it is larger than the threshold value, the scale factor’s parameter is estimated in the current frame.

## 4. Experimental Results

### 4.1. Datasets and Experimental Setup

**Figure 6:**
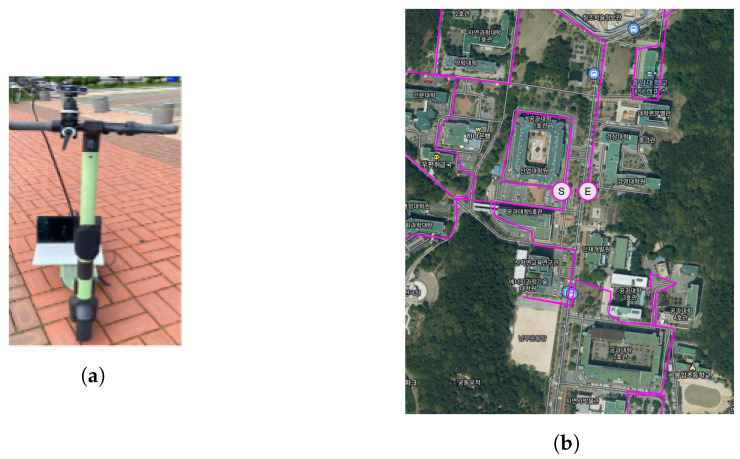
CNU datasetacquired using Intel RealSense D435. (**a**) Scooter, (**b**) a part of university map and path for collecting data(magenta line, S and E mean start and end point respectively).

The directly collected dataset and open datasets were used to verify the performance of the method proposed in this paper. The CNU dataset was collected using an Intel RealSense D435 (baseline: 50 mm) on Chungnam National University(CNU), Daejeon in South Korea (Figure 6). The UASOL dataset [13] was collected using ZED (baseline: 120 mm) on a university campus in Spain. For the RADIATE dataset [14], the same camera as the UASOL dataset was used, but the images were captured in urban and suburban areas in the United Kingdom under various lighting and weather conditions.

The CNU dataset provides distance data up to 10 m, whereas the UASOL dataset does within 20 m. The CNU and UASOL data do not give a LiDAR depth to provide an accurate physical distance. In contrast, the RADIATE [14] dataset provides LiDAR depths in adverse weather conditions such as rain, fog, and snow with left and right color images but not SD. Furthermore, as the quantitative evaluation of the data collected using a short baseline camera equipped with a precise LiDAR sensor is in progress as a future study, we used the stereo depths of UASOL and RADIATE LiDAR’s within 20 m to check the performance of the proposed method. Furthermore, we introduce the qualitative results for the UASOL and CNU data containing SDs. The proposed method was implemented with CUDA 11.02 and Python 3.8.5 on an Intel Xeon(R) CPU E5-2609@1.7 G (8 cores), 64 G memory, Nvidia RTX2080Ti, and docker 20.10.5.

### 4.2. Performance Evaluation

For the indirect quantitative evaluation of the proposed method, we use the trained BTS and AdaBins models and apply them to UASOL and RADIATE datasets without any modification. As a result, we verify that the pretrained model can be used as it is without collecting new data and training a model again in new environments. To do this, we compare the differences between MD and GT, and FD and GT, respectively, according to the metrics in Table 1.

We divide the distances into three categories to evaluate the proposed method’s performance using the UASOL and RADIATE datasets: <10 m, 10–15 m, and 15–20 m. In each area, the depth estimation results are shown in Table 2. To estimate MD, we employed AdaBins and BTS models trained on the KITTI dataset. These results indicate that two models do not perform well in situations other than those in which the dataset was acquired for training. The proposed method, on the other hand, has smaller depth estimation errors than the two models. The proposed method estimates depths more accurately than the two models without retraining, according to depth estimation results using the CNU dataset (Table 3).

**Figure 7:**
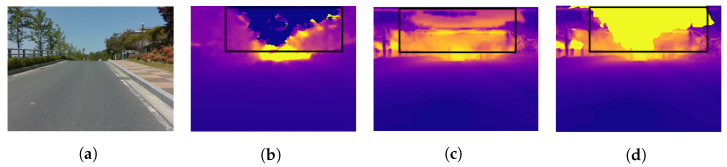
Sensing range extension result. (**a**) RGB, (**b**) stereo depth with sky in black box, (**c**) depth fusion without sky using semantic segmentation, and (**d**) depth fusion (Purple is near yet yellow is far distance).

We use the CNU and UASOL datasets to qualitatively demonstrate that the proposed method expands the recognition range of SBSC. To exclude the parts belonging to the sky region in the process of estimating the scale factor’s parameter, we used the DeepLab v3 model [17] trained using the CityScapes dataset [18] with the backbone of MobileNet v2 [19]. Figure 7 shows examples of the results of expanding the sensing range of the stereo camera that has a short baseline using the proposed method. In the SD shown in Figure 7b, the depth of the close region is estimated accurately, but the depth of a far region is not estimated. As shown in Figure 7c, the proposed method can estimate the depth to a further region than the SD. The regions marked with a black rectangle in Figure 7b–d, respectively, are the sky. Because of the lack of texture in the SD calculation process, ghosts appear in the sky region. In the case of MD, there are more ghosts in the sky than in the case of SD. Figure 7d shows the result of the applied sky removal using semantic segmentation to the proposed method. The proposed method and semantic segmentation can be used to estimate the depth even for a long distance, and the ghosts appearing in the sky region can also be removed.

**Figure 8:**
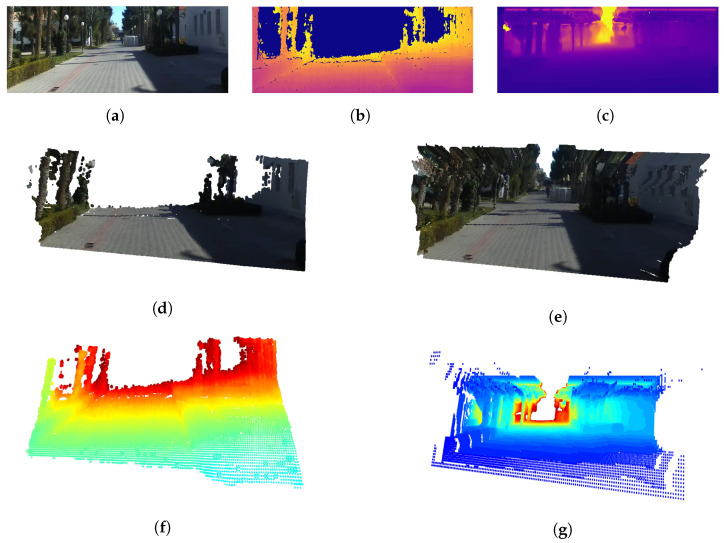
3D reconstruction results using UASOL dataset. (**a**) RGB, (**b**) stereo depth, (**c**) mono depth, (**d**) stereo depth mesh, (**e**) depth fusion mesh, (**f**) stereo depth voxel, and (**g**) depth fusion voxel.

**Figure 9:**
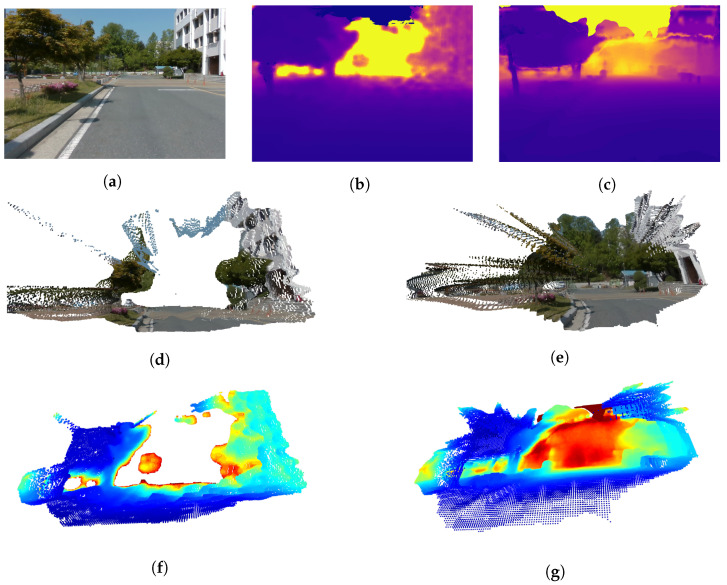
3D reconstruction results using CNU dataset. (**a**) RGB, (**b**) stereo depth, (**c**) mono depth, (**d**) stereo depth mesh, (**e**) depth fusion mesh, (**f**) stereo depth voxel, and (**g**) depth fusion voxel.

The 3D visualization results of the depths estimated using the proposed method using the UASOL and CNU datasets, respectively, are shown in Figure 8 and Figure 9. They show that the proposed method can be used to estimate the depth of a long-distance by expanding the sensing range of the SBSC. Figure 8d,f, and Figure 9d,f are the results of visualizing the SD that the SBSC obtained as the 3D mesh and 3D voxel. They confirm the limitation that the SD obtained by the SBSC can be used to recognize the structure of the environment in a close region. Figure 8e,g and Figure 9e,g show the results of visualizing the depths estimated using the proposed method as the 3D mesh and 3D voxel. They show that if the proposed method is used, the structure of the environment can be recognized, even for the region of a long distance. Furthermore, it is confirmed that semantic segmentation removes the ghosts in the sky region, resulting in no unknown obstacles.

**Figure 10:**
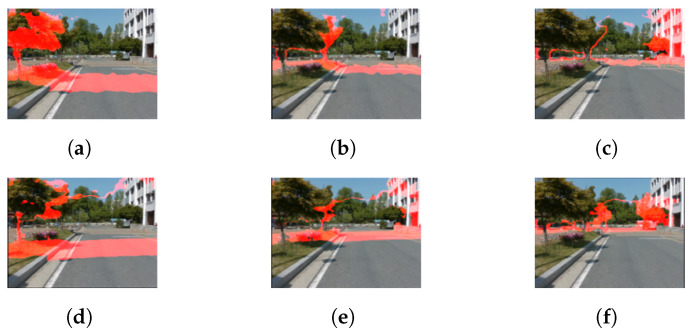
Depth estimation results using CNU dataset. (**a**) Stereo depth 5 to 10 m, (**b**) stereo depth 10 to 20 m, (**c**) stereo depth 20 to 40 m, (**d**) depth fusion (5 to 10 m), (**e**) depth fusion (10 to 20 m), and (**f**) depth fusion (20 to 40 m).

**Figure 11:**
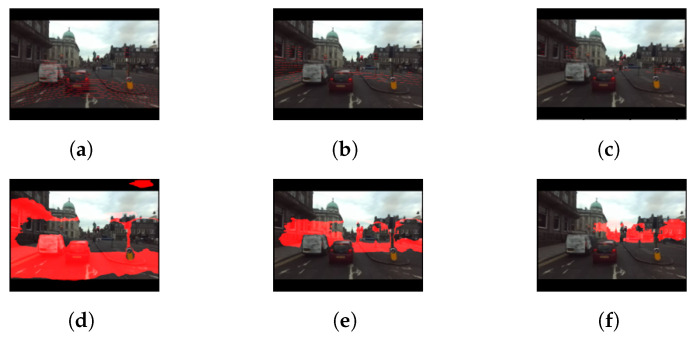
Depth estimation results using RADIATE dataset. (**a**) LiDAR depth 5 to 10 m, (**b**) LiDAR depth 10 to 20 m, (**c**) LiDAR depth 20 to 40 m, (**d**) depth fusion (5 to 10 m), (**e**) depth fusion (10 to 20 m), and (**f**) depth fusion (20 to 40 m).

**Figure 12:**
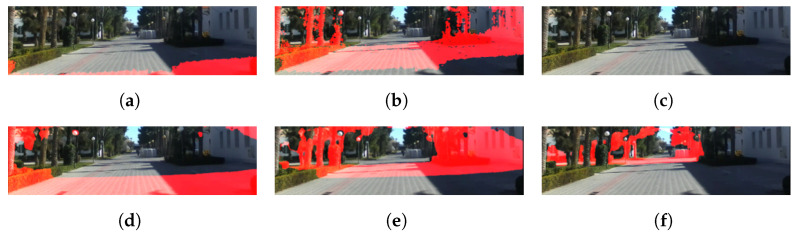
Depth estimation results using UASOL dataset. (**a**) Stereo depth 5 to 10 m, (**b**) stereo depth 10 to 20 m, (**c**) stereo depth 20 to 40 m, (**d**) depth fusion (5 to 10 m), (**e**) depth fusion (10 to 20 m), and (**f**) depth fusion (20 to 40 m).

In Figure 10, Figure 11 and Figure 12, the structure of the environment outside the range that the SBSC can be sensed using the proposed method. The red regions represent the point groups within certain ranges (5–10 m, 10–20 m, 20–40 m).

### 4.3. Computational Performance

Based on the strategy proposed in Section 3.4, we measured the performance of the proposed method by not having to estimate the scale factor’s parameter for every frame. Since the environment structure may vary, the frequency of reusing the scale factor parameter obtained in the previous frame may vary. Therefore, the RADIATE and KITTI datasets were also used, in addition to the UASOL and CNU datasets for the test. Table 4 shows the result for each dataset with 0.5 m as the threshold value *e*. The frequency of parameter estimation for the scale factor varies based on the dataset, according to this statement. Because dissimilar images were used in nine places on campus with different landscapes, the UASOL dataset took the longest time to estimate the scale factor parameter, causing many structural changes in the dataset. The other three datasets, on the other hand, do not show major changes in the environment structure because they are relatively constant at a campus or city center. As a result, once the scale factor parameter has been computed, it can be used over multiple frames. The computing time was mostly used to estimate the monocular depth during the entire process of evaluating depths. If faster computing speed is required, a method, such as FastDepth [20], that has faster processing speed than BTS and AdaBins can be used.

## 5. Conclusions and Future Works

To expand the SBSC’s sensing range, this paper proposes a method that takes the SD of a limited range obtained from the SBSC and calculates the scale factor of the MD obtained using the MDE to combine the two depth estimations in an FD. If it is used, we can possibly recognize the environment up to a long-distance using the SBSC. An additional advantage of ours is that the trained MDE model can be used without retraining for different data in different environments. Usually, if the MDE model is used without retraining in an environment different from the trained, the relative depth may differ significantly from the actual one. Usually, it brings tedious data manipulation (acquisition, annotation, preprocessing, etc.) with multi-channel LiDAR and SBSC. However, the proposed method can calibrate the MD by estimating the scale factor’s parameter.

Table 5 shows the pros and cons between the various methods and ours for obtaining distance data that can be importantly utilized by small platforms.

The method can be used to improve mapping, path planning, or obstacle avoidance performance by recognizing the environment, even for a long distance, on a small platform where a sensor with a large size and large power consumption, such as LiDAR, cannot be installed.

Unfortunately, we could not construct a dataset capable of quantitatively verifying the depth estimation accuracy of it and performed some qualitative validations with limited quantitative ones. In a future study, we will build a dataset to validate the depth estimation accuracy more quantitatively. Furthermore, we plan to apply it to path planning.

## Figures and Tables

**Table 1:** Evaluation metrics.

Errors	Equations
Average relative error (AR)	$\frac{1}{\left|N\right|}\sum_{\hat{X}\in N}\left|{\hat{X}}_{s}-X_{s}\right|/X_{s}$
Squared relative difference (SR)	$\frac{1}{\left|N\right|}\sum_{\hat{X}\in N}\left(\right|{\hat{X}}_{s}-X_{s}{\left|\right)}^{2}/X_{s}$
Root mean squared error (RS)	$\sqrt{\frac{1}{\left|N\right|}\sum_{\hat{X}\in N}\left(\right|{\hat{X}}_{s}-X_{s}{\left|\right)}^{2}/X_{s}}$
Average Log 10 error (L10)	$\frac{1}{\left|N\right|}\sum_{{\hat{X}}_{s}\in N}\left|\log_{10}{\hat{X}}_{s}-\log_{10}X_{s}\right|$

**Table 2:** Comparison of performances on UASOL and RADIATE datasets within 20 m.

DB	Network	GT vs.	Evaluation Results											
			AR			SR			RS			L10		
			D10 ^1^	D15 ^2^	D20 ^3^	D10	D15	D20	D10	D15	D20	D10	D15	D20
UASOL	BTS	MD	0.35	0.45	0.56	1.39	4.54	7.66	2.97	6.64	10.49	0.17	0.25	0.29
		FD	0.14	0.33	0.47	0.32	2.31	5.61	1.31	4.67	8.76	0.05	0.16	0.26
	AdaBins	MD	0.30	0.43	0.47	1.11	3.02	4.91	2.57	5.74	8.78	0.12	0.21	0.26
		FD	0.13	0.32	0.44	0.31	1.82	4.28	1.26	4.40	8.04	0.04	0.16	0.24
RADIATE	BTS	MD	0.56	0.59	0.55	2.76	5.50	7.35	4.45	8.25	11.01	0.19	0.19	0.17
		FD	0.09	0.19	0.27	0.12	0.68	1.68	0.89	2.89	5.19	0.03	0.08	0.12
	AdaBins	MD	0.44	0.506	0.46	1.88	4.06	4.92	3.64	7.04	8.99	0.16	0.16	0.13
		FD	0.1	0.14	0.21	0.16	0.53	1.27	1.01	2.50	4.56	0.022	0.04	0.07

^1^ D10: under 10 m, ^2^ D15: [10–15] m, ^3^ D20: [15–20] m.

**Table 3:** Comparison of performances on CNU dataset within 10 m.

DB	Network	ST vs. ${}^{1}$	Evaluation Metrics			
			AR (D10) ^2^	SR (D10)	RS: (D10)	L10 (D01)
CNU	BTS	MD	0.653	3.85	5.186	0.206
		FD	0.153	0.37	1.481	0.043
	AdaBins	MD	0.653	4.106	5.302	0.202
		FD	0.147	0.294	1.013	0.042

^1^ ST: RealSense camera depth. ^2^ D10: under 10 m.

**Table 4:** Computational performance.

Network	Measure	CNU (640 × 480)	UASOL (1216 × 352)	RADIATE (640 × 480)	KITTI (1216 × 352)
BTS	# of SFPE ^1^/sample	4/1760	74/205	7/719	0/433
	SFPE time (μ/σ) (s)	0.014/0.004	0.061/0.055	0.013/0.009	0.020/0.004
	MDE time (μ/σ) (s)	0.159/0.004	0.432/0.010	0.160/0.007	0.215/0.007
	Total (s)	0.173	0.493	0.173	0.235
AdaBins	# of SFPE/sample	9/1760	66/205	0/719	0/433
	SFPE time (μ/σ) (s)	0.014/0.005	0.057/0.057	0.012/0.003	0.019/0004
	MDE time (μ/σ) (s)	0.201/0.002	0.230/0.004	0.203/0.002	0.227/0.002
	Total time (s)	0.215	0.287	0.215	0.246

^1^ SFPE: Scale Factor Parameter Estimation.

**Table 5:** Comparison of depth acquisition methods for small mobile robot.

Depth Methods	Pros	Cons
LiDAR	accurate	sparse, high cost and energy, relatively more mounting space
Stereo ^1^	dense, relatively low cost and energy, less mounting space	accurate only within short distance ^3^
Monocular ^2^	same with stereo depth	require data manipulation ^4^ and retraining of CNNs if not like with train environments
Proposed Method	same with stereo depth, no manipulation, and retraining	relatively inaccurate if used in the train environments compared with MDE

^1^ Short baseline camera. ^2^ Based on CNNs. ^3^ Dependent upon its specification. ^4^ Data manipulation: gathering, annotation, proprocessing, etc.

## Data Availability

Not applicable.
